# “NanoDel”: identification of large-scale mitochondrial DNA deletions using long-read sequencing

**DOI:** 10.1093/bioinformatics/btag684

**Published:** 2026-09-15

**Authors:** C Fearn, J Poulton, C Fratter, C Oliva, C Griguer, R A Baldock, S C Robson, R E McGeehan

**Affiliations:** School of Medicine, Pharmacy and Biomedical Sciences, Institute of Life Sciences and Healthcare, University of Portsmouth, Portsmouth, United Kingdom; Nuffield Department of Women’s Reproductive Health, University of Oxford, Oxford, United Kingdom; Oxford Genetics Laboratories, Oxford University Hospitals NHS Foundation Trust, Oxford, United Kingdom; Free Radical and Radiation Biology Program, University of Iowa, Iowa City, Iowa, United States; Free Radical and Radiation Biology Program, University of Iowa, Iowa City, Iowa, United States; School of Medicine, Pharmacy and Biomedical Sciences, Institute of Life Sciences and Healthcare, University of Portsmouth, Portsmouth, United Kingdom; School of Medicine, Pharmacy and Biomedical Sciences, Institute of Life Sciences and Healthcare, University of Portsmouth, Portsmouth, United Kingdom; Centre for Enzyme Innovation, Institute of Life Sciences and Healthcare, University of Portsmouth, Portsmouth, United Kingdom; School of Medicine, Pharmacy and Biomedical Sciences, Institute of Life Sciences and Healthcare, University of Portsmouth, Portsmouth, United Kingdom

## Abstract

**Motivation:**

Traditional methods for detecting large-scale mitochondrial DNA (mtDNA) deletions (LSMDs) in cells present challenges, i.e. requiring a priori information, high DNA inputs, and are not always sensitive and/or quantitative. Mitigation can be achieved through high-throughput DNA sequencing using e.g. Illumina and Oxford Nanopore Technologies (ONT), in combination with LSMD breakpoint identification and quantification using bioinformatics. Splice-aware RNA alignment tools increase the sensitivity for detecting LSMD breakpoints compared with DNA aligners. Long-read sequencing (LRS) also offers potential advantages over short-read sequencing (SRS), e.g. greater read lengths and capturing variants on single reads. Here we aimed to capture the benefits of both a splice-aware alignment tool and LRS.

**Results:**

We developed “NanoDel,” a LRS pipeline, to sensitively and accurately detect cellular LSMDs. Using artificial datasets, “NanoDel” was more sensitive and accurate than other pipelines. In samples diagnosed with mitochondrial disease, it identified both known and previously uncharacterised (including mixtures) of LSMDs, without a priori information. Analysis of selected LSMDs revealed proximity to repeat, putative G-quadruplex motifs, and the “contact zone.” Together with occurrence in a range of healthy and pathological tissues, indicates potential for a shared vulnerability landscape in mtDNA, shaped by sequence motifs and structural constraints. This proof-of-concept study shows that “NanoDel” combined with one-amplicon LR-PCR offers a robust strategy for detecting LSMDs across a variety of cell/tissue samples. Applying “NanoDel” to a larger and broader range of samples would confirm this, yielding new mechanistic insights into LSMD formation, and further our understanding of mtDNA instability in the future.

**Availability and implementation:**

"NanoDel" is an open-source pipeline freely available on GitHub at https://github.com/uopbioinformatics/NanoDel and permanently archived via Zenodo (DOI: 10.5281/zenodo.20119070).

## 1 Introduction

Mitochondria are cellular organelles that have their own double-stranded, supercoiled genome; mitochondrial DNA (mtDNA). MtDNA has 38 loci, including 13 protein coding genes, 2 rRNAs, 22 tRNAs and the control region (D-loop). The D-loop contains regulatory elements involved in mtDNA transcription and replication. Together, with approximately 70 nuclear-encoded proteins, the 13 mtDNA-encoded subunits form the multi-protein mitochondrial respiratory chain (MRC) complexes (I-IV) and ATP synthase (complex V) that are collectively known as the oxidative phosphorylation (OXPHOS) system. This system is central to ATP generation and cellular metabolism (reviewed in Vafai and Mootha 2012). Direct and/or indirect alterations in the mitochondrial genome can impact the function of the OXPHOS system, and therefore cellular energy generation, contributing to disease (for examples, see Table S1, available as supplementary data at *Bioinformatics* online). Large-scale mtDNA deletions (LSMDs), which are direct alterations to mtDNA, result in genes crucial for cellular energy production via OXPHOS being removed and, therefore, primary mitochondrial disease.

Several traditional methods, such as Southern blotting (Moraes *et al.* 1989, Santorelli *et al.* 1996), PCR (Schon *et al.* 1989) and Sanger sequencing (Johns *et al.* 1989, 1989, Mita *et al.* 1990, Poulton *et al.* 1989), have been used historically to detect LSMDs. However, the accurate breakpoint and heteroplasmy determinations using these techniques is challenging, requiring a priori breakpoint information, and high DNA inputs. Often such methods also lack sensitivity, are not completely quantitative, and can be labour intensive. One way to identify the breakpoints where multiple unknown LSMDs are present would be to plasmid clone and select all molecules prior to Sanger sequencing, however this is even more labour intensive (Schon *et al.* 1989).

A useful and sensitive alternative for identifying unknown LSMDs is by LR-PCR to amplify whole mtDNAs, followed by high-throughput DNA sequencing using platforms from e.g. Illumina (Hjelm *et al.* 2019) or Oxford Nanopore Technologies (ONT), and subsequent breakpoint identification and quantification using bioinformatic tools. Indeed, in 2019, Hjelm *et al.* published a pipeline that combines the LR-PCR of whole mtDNAs, as single amplicons, followed by Illumina sequencing, and a splice-aware RNA alignment tool for breakpoint identification and quantification. Hjelm’s pipeline increased the sensitivity of detecting true positives compared to approaches like MitoDel (Bosworth *et al.* 2017) when using traditional DNA alignment tools (e.g. Burrows-Wheeler aligner) by around 60% (Hjelm *et al.* 2019). While examples of LMSDs detection by ONT sequencing exist, traditionally these have not used LR-PCR or a splice-aware aligner (Frascarelli *et al.* 2023, Vandiver *et al.* 2023). However, employing LR-PCR and ONT sequencing for LSMD detection could offer additional advantages over short read (e.g. Illumina) sequencing, including reduced amplification of nuclear mitochondrial DNA segments (NUMTs) and longer individual read lengths (>1kb vs. <300bp) that span breakpoints, meaning a single read can represent an entire variant. This allows for the possibility of analysing variants, including SNVs, in tandem on the same read.

With improvements to sequencing chemistry and base-calling models now achieving quality scores above Q20, ONT is an attractive alternative to Illumina for the accurate breakpoint identification and quantification of LSMDs. However, as no comparable ONT pipeline to that of Hjelm *et al.* (2019) existed for LSMDs around the time it was published, we developed “NanoDel.” This pipeline endeavours to amplify whole mtDNAs as either one or two amplicons by LR-PCR, followed by ONT sequencing, and a bespoke combination of bioinformatic tools for accurate breakpoint identification and quantification in cells/tissues.

In this study we first compared the ability of four pipelines to determine LSMDs breakpoints at different heteroplasmy levels using two artificial data sets. The four pipelines included two that use long-read sequences either in combination with a splice-aware RNA aligner (“NanoDel,” Pipeline 1, this study) or a DNA aligner (“Wood ONT,” Pipeline 3, Wood *et al.* 2019), and two that use short read Illumina sequences either in combination with a splice-aware RNA aligner (“Splice-Break,” Pipeline 2, (Hjelm *et al.* 2019)) or a DNA aligner (“Wood Illumina,” Pipeline 4, (Wood *et al.* 2019)). Second, we directly compared the two different ONT-pipelines (“NanoDel” and “Wood ONT”) to understand the impact of the selected alignment strategy, and the two LR-PCR approaches (one and two-amplicon) to determine known LSMDs in Kearns-Sayre syndrome (KSS) tissues. Third, we compared the LSMDs information generated by “NanoDel” and one-amplicon PCR against LSMDs information obtained diagnostically (using traditional methods) for additional patients with mitochondrial disease. This provides a comprehensive benchmarking of the power of “NanoDel” for exploring unknown LSMDs from clinical samples, allowing for fine-tuned understanding of the impact of mtDNA instability on disease in the future.

## 2 Methods

### 2.1 Patient-derived tissues, cell cultures and DNA extraction

Three total DNA samples extracted from the kidney, cerebellum, and muscle of a KSS patient of known LSMD composition determined by Southern blotting (Table 1) were provided by Professor Joanna Poulton at the Nuffield Department of Women’s and Reproductive health (Patient 1 in (Poulton *et al.* 1989, 1993, Poulton *et al.* 1995), University of Oxford. Additionally, DNA samples from muscle (muscle_RNASEH1, muscle_TWNK and muscle_TYMP) and cultured cells (cells_nd and fibroblasts_nd) were provided by Professor Joanna Poulton and by Carl Fratter at the Oxford Genetics Laboratories (OGL). These DNA samples were prepared and de-identified prior to transfer to our laboratory.

**Table 1:** Cell culture and patient details.

BioSample name	Accession No.	Source (Institute/Company)	Diagnosis	Source (tissue)	Deletion type	Breakpoints, how determined	Heteroplasmy, how determined	Ref
UOP_1_muscle_RNASEH1	SAMN53383571	Oxford Medical Genetics Laboratories	Primary genetic cause = AR disease due to RNASEH1 variants	Muscle	Multiple	n.d. ∼8.6 kb (by Southern blot)	n.d.	This study
UOP_2_cells_nd	SAMN53383572	Oxford Medical Genetics Laboratories	n.d.	Cultured cells	Single	m.8649_16084del (by Sanger sequencing)	∼72–76% (by qPCR)	This study
UOP_3_fibroblasts_nd	SAMN53383573	Oxford Medical Genetics Laboratories	n.d.	Fibroblasts	Single	m.8483_13459del (common deletion)(by Sanger sequencing)	n.d.	This study
UOP_4_muscle_TYMP	SAMN53383574	Oxford Medical Genetics Laboratories	Primary genetic cause = AR disease due to TYMP variants	Muscle	Multiple	n.d. (by Southern blot)	n.d.	This study
UOP_5_muscle_TWNK	SAMN53383575	Oxford Medical Genetics Laboratories	Primary genetic cause = AD disease due to TWNK variant	Muscle	Multiple	n.d. ∼2.9 kb (by Southern blot)	n.d.	This study
UOP_6_muscle_KSS	SAMN53383576	University of Oxford	KSS	Muscle	n.a.	m. 6130–15056 (by Southern blot)*	47% (by Southern blot)	Poulton *et al.* 1989, 1993, 1995
UOP_7_cerebellum_KSS	SAMN53383577	University of Oxford	KSS	Cerebellum	n.a.	m. 6130–15056 (by Southern blot)*	23% (by Southern blot)	Poulton *et al.* 1989, 1993, 1995
UOP_8_kidney_KSS	SAMN53383578	University of Oxford	KSS	Kidney	n.a.	m. 6130–15056 (by Southern blot)*	54% (by Southern blot)	Poulton *et al.* 1989, 1993, 1995
UOP_9_cells_143B	SAMN53383579	Kerafast		Osteosarcoma				This study
UOP_10_cells_Rho-zero	SAMN53383580	Kerafast		Osteosarcoma (143B with mtDNA eliminated)				n.a.
UOP_11_cells_SC1800-1	SAMN53383581	ScienCell Research Laboratories		Adult, cerebral cortex, non‐neoplastic astrocyte				Lloyd *et al.* 2015
UOP_12_cells_SC1800-2	SAMN53383582	ScienCell Research Laboratories		Adult, cerebral cortex, non‐neoplastic astrocyte				This study

KSS, Kearns-Sayre Syndrome; AR, autosomal recessive; AD, autosomal dominant; RNASEH1, ribonuclease H1, mtDNA replication/repair; TWNK, Twinkle, mtDNA helicase; TYMP, thymidine phosphorylase, mtDNA replication and maintenance; n.d., not determined; n.a., not applicable. *these samples have also been shown to contain a duplication m. 15056–6130 and deletion dimers.

The original tissues were surplus to the quantity required for diagnosis, with the KSS patient tissues donated for research on the 1 January 1990 with permission from the patient’s family and surgeon, and the remaining patient tissue donated to research between 19 May 2005 and 13 December 2024 with written informed consent from all participants and with the approval of the United Kingdom National Research Ethics Service (South Central-Berkshire, #REC 20/04, IRAS 162181). Previous observations, i.e. LSMD composition determined by a range of different techniques following diagnosis at the OGL, were made available for these samples following sequencing analysis to prevent bias (Table 1). Details of cell lines used as mtDNA positive and negative amplification controls are also provided (Table 1). Total DNA was extracted from all cell culture samples using the QIAmp DNA mini kit as per the manufacturer’s instructions (Qiagen, 51304).

### 2.2 Patient mitochondrial genome sequence generation

Whole mtDNAs were amplified from patient DNA in either two overlapping amplicons (Keatley *et al.* 2019, Lloyd *et al.*  2012, 2015) or as a single amplicon (Hjelm *et al.* 2019) by LR-PCR using human mtDNA-specific primers from total DNA (Table S2, available as supplementary data at *Bioinformatics* online). For the two amplicon approach, the primers were CytbF and Human Long R (binding mt-cyb and mt-co1 at nucleotide positions (nps) 15,029–15,049 and 7053–7078, respectively) for the first amplicon, and HumanLongF and CytbR (binding mt-co1 and mt-cyb at nps 6578–6603 and 15,731–15,750, respectively), for the second amplicon. For one-amplicon approach, the primers were Alt Human LR-PCR F and R (binding the mt-d-loop at nps 16,523–16,543 and 16,199–16,219, respectively). This resulted in a 304 bp portion of the mt-d-loop that was not amplified, including 5 bp of mt-csb-i (nps ∼16,518–16,540) and 164 bp of Hypervariable Region 1 (mt-hv1, nps 16,024–16,383). Breakpoints occurring in or spanning this gap would not have been detected using our approach (Fig. S1, available as supplementary data at *Bioinformatics* online).

Briefly, Nanopore libraries were prepared from either two or single LR-PCR amplicons using the Native Barcoding Kit 96 V12 library kit (ONT, NBD112.96), barcoded using the NBD112.96 kit and the NEB Blunt T/A Ligase MasterMix (NEB, M0367S), multiplexed and then cleaned using AMPure XP beads (Beckman Coulter, A63880). Adapter sequences were ligated to the multiplexed DNA sample using the NEBNext Quick Ligation module (New England Bioscience, E6056S). The prepared, washed and quantified DNA library was then loaded to an R10.4 flow cell and sequenced for 72 hours on an Oxford Nanopore Technologies GridION X5. Full details of the one- and two-amplicon LR-PCR, library preparation and sequencing are provided in the Supplementary materials and methods, available as supplementary data at *Bioinformatics* online.

### 2.3 Bioinformatics, artificial data generation, alignment and LSMD analyses

First and briefly, two styles of artificial FASTQ format raw sequence data files were generated using VISOR (v1.1, (Bolognini *et al.* 2020) in: (1) an ONT style with a longer average read length of ∼8kb and a “Nanopore 2020” style error model, and (2) an Illumina style with 250 bp length reads and an “Illumina” style error model. Each style contained either controlled quantities of reads designed to emulate a single deletion (mtDNA 4977) at different levels; i.e. 75%, 50%, 25%, 5%, 1% and 0.25% of reads (artificial data 1) or multiple deletions (m.8471-13449del, m.6620_8440del, m.3451_5500del and m.12538_14572del) at a fixed quantity (10%) of reads (artificial data 2). These files were then used to evaluate and compare the efficacy of the four separate alignment and deletion calling strategies outlined above (Pipelines 1–4). Second, raw reads from the patient mtDNA LR-PCR libraries (one- and/or two-amplicon) were base-called and demultiplexed (including barcode trimming) using Guppy (v6.2.7 and v6.3.9) and transferred to a local high-performance computing cluster running on Ubuntu (v20.04.5). Base-called reads were initially filtered to remove reads of <20bp in length or a Q-score <10 using NanoFilt (v2.8.0, (De Coster *et al.* 2018) prior to processing through the two best performing pipelines: “NanoDel” (Pipeline 1, Fig. 1) and “Wood ONT” (Pipeline 3). In this study all reads (artificial and patient) were aligned to the rCRS reference sequence (NC_012920.1, (Andrews *et al.* 1999, Bolognini *et al.* 2020). Full details of the artificial data generation, alignment and deletion calling strategies, including all read quality control, software, benchmarking and filtering are given in the Supplementary materials and methods, available as supplementary data at *Bioinformatics* online.

**Figure 1:**
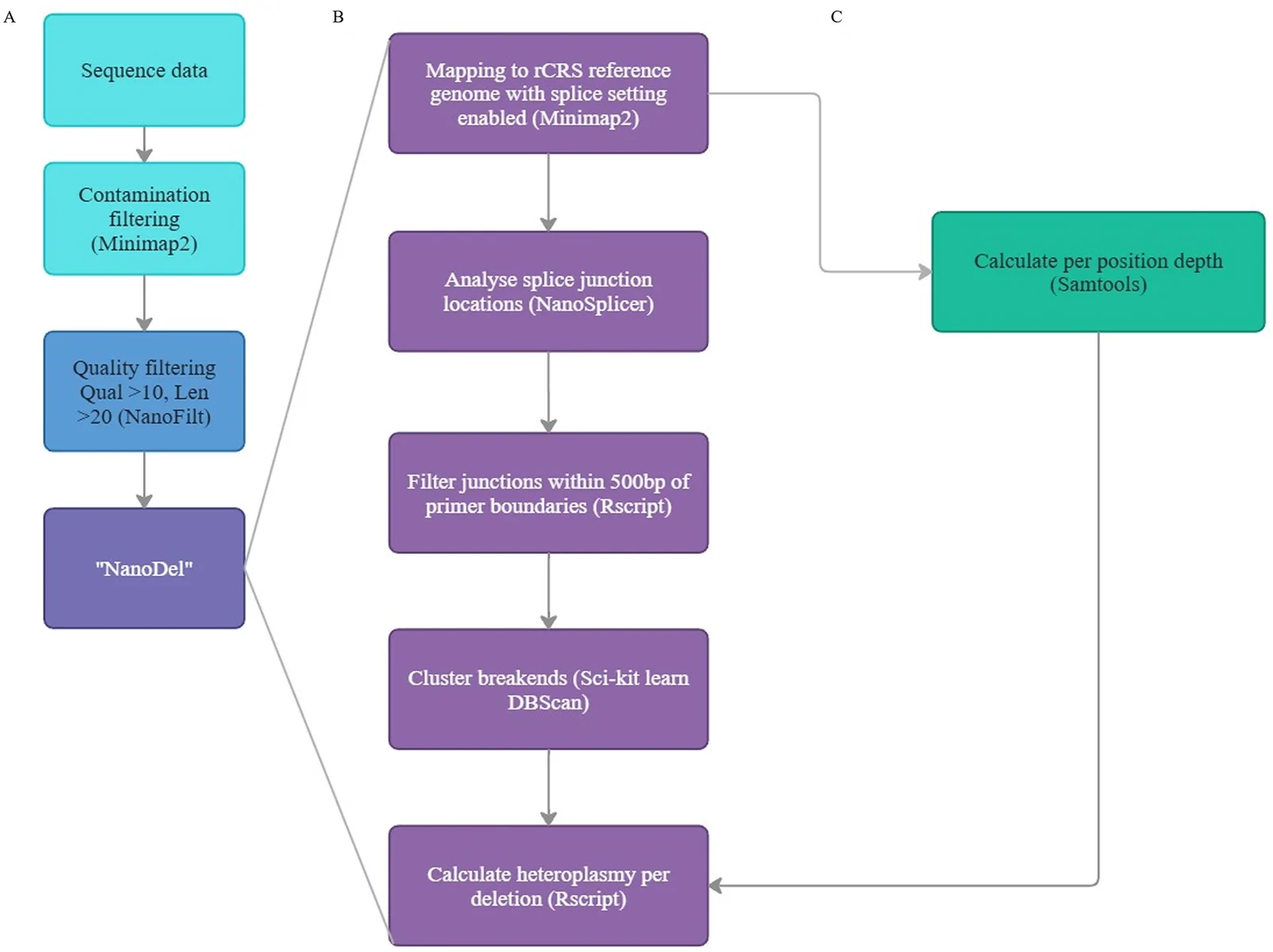
Flow diagram of large-scale mtDNA deletions using Pipeline 1 (“NanoDel”) developed for this study. (A) Pre-processing of raw reads, (B) running “NanoDel” through mapping to reference, extracting splice junction locations, filtering junctions within 500 bp of primer boundaries, clustering breakends within 10 bp of each other, and finally relative read% for each deletion was calculated using (C) mean coverage benchmark (i.e. the depth output between positions m.500_1000).

### 2.4 Repeat and G4 sequence, previous tissue/disease association and statistical analyses

Sequences flanking the 5’- and 3’-breakpoints of the mitochondrial disease patient LSMDs occurring at ≥50% of reads detected in the filtered “NanoDel” data (Pipeline 1) were analysed for repeat sequences and their potential to form G-quadruplexes (G4s) using QGRS mapper (Kikin *et al.* 2006) and AlphaFold 3 (AF3, Abramson *et al*. 2024).

Previous tissue and/or disease associations of the LSMDs, both “NanoDel” (Pipeline 1) and adjusted positions (Table S5, available as supplementary data at *Bioinformatics* online), and 10 bp flanking either side were investigated using the Mitobreak database, which contains 1369 human mtDNA deletions previously described in the literature (Damas *et al.* 2014).

Statistical analyses of post-alignment read metrics were conducted using GraphPad Prism (v9.4.1). The normality of each metric for each pipeline was assessed using the Shapiro-Wilk test (Shapiro and Wilk 1965). Where assumptions of normality were met, a Brown-Forsythe and Welch ANOVA was employed to model differences between the approaches. If the assumption of normality was violated, the non-parametric Kruskal-Wallis test (Kruskal and Wallis 1952) was used instead. Further post-hoc pairwise comparisons were made using either Welch’s *t*-test or the non- parametric Mann-Whitney test, respectively. Full details on the repeat and G4 analyses are given in the Supplementary materials and methods, available as supplementary data at *Bioinformatics* online.

## 3 Results

The pipeline developed for this study, “NanoDel” (Pipeline 1), was tested against three other pipelines using two artificial datasets to compare the performance in detecting the percentage of reads within which each LSMD occurs, and its location. “NanoDel” offered the best balance between accuracy, sensitivity and specificity when identifying the heteroplasmy and breakpoint location, both as a single deletion (mtDNA4977) in artificial data set 1, and as mixed deletions in artificial data set 2 (Figs 2–3, and Supplementary Results, available as supplementary data at *Bioinformatics* online). Due to the performance of “NanoDel” (Pipeline 1) in detecting LSMDs. within the artificial data, it was then employed to detect LSMDs and determine their breakpoints in two small mitochondrial disease patient cohorts (Table 1) following both one- and two-amplicon LR-PCR. MtDNA breakpoints were also determined following LR-PCR amplification in these samples using “Wood ONT” (Pipeline 3) for comparison. LSMDs were observed in clinically diagnosed mitochondrial disease samples on agarose gels following different LR-PCR approaches, revealing single or multiple deletion types (Fig. S2, and Supplementary results, available as supplementary data at *Bioinformatics* online). Post-alignment, “NanoDel” yielded lower mapping %, (Fig. 4A), error rates (Fig. 4B and G vs. L and Q), higher mean coverage (Fig. 4C) and higher maximum read lengths (Fig. 4E) compared to “Wood ONT” with Canu (Pipeline 3). Furthermore, “NanoDel” (Pipeline 1) and “Wood ONT” without Canu (Pipeline 3) had increased mapping rate (Fig. 4F and P) but a higher error rate (Fig. 4G and Q) following one- rather than two-amplicon LR-PCR (see also Table S3 A-B, and Supplementary results, available as supplementary data at *Bioinformatics* online).

**Figure 2:**
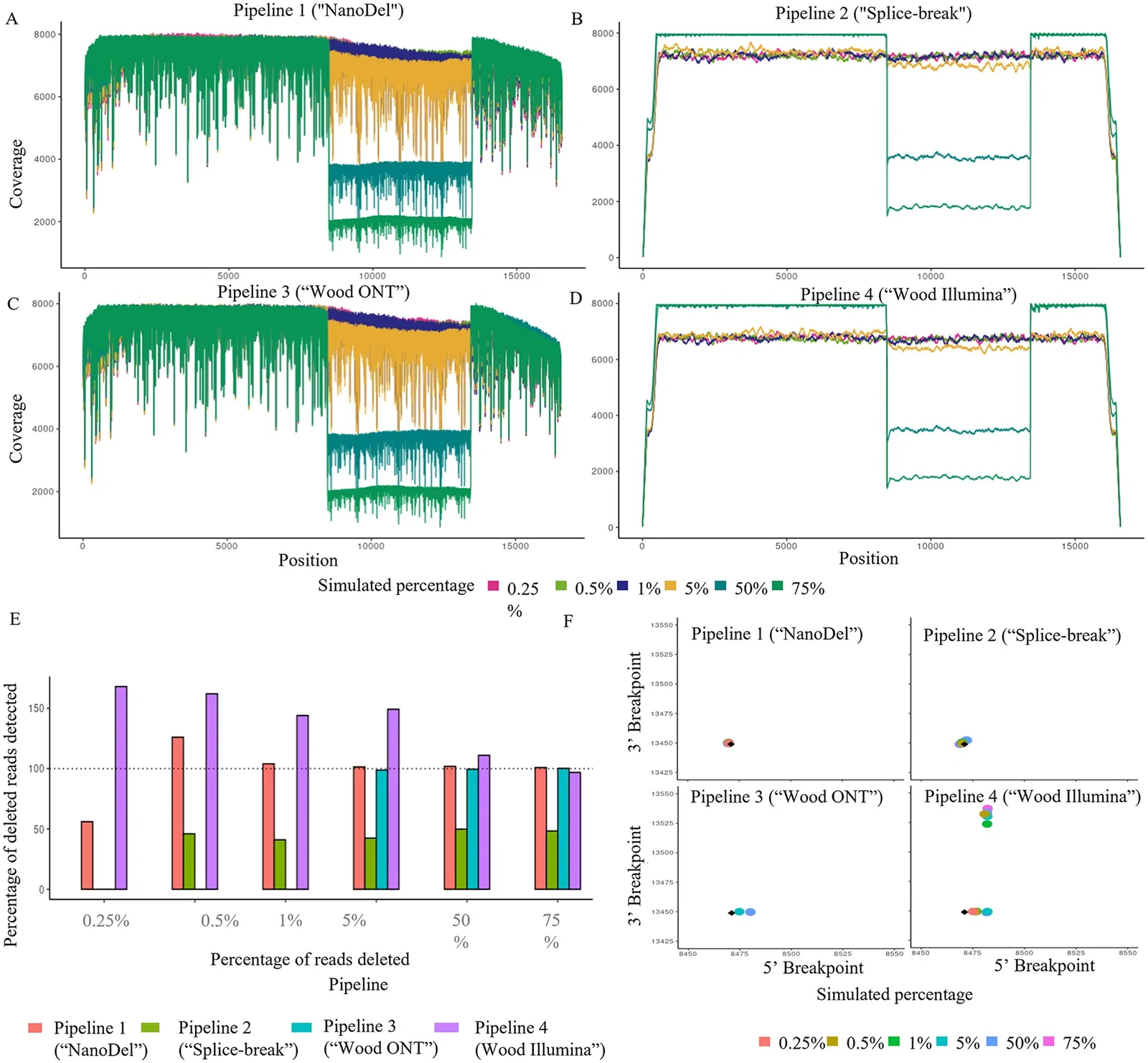
Analysis of artificial data set 1 that contains a mixture of the mtDNA4977 (m. 8471_13449del) and wild type mtDNA at different ratios. (A–D) The per-position levels of coverage generated by Pipeline 1 (“NanoDel”), Pipeline 2 (“Splice-break”), Pipeline 3 (“Wood ONT”), and Pipeline 4 (“Wood Illumina”), respectively. (E) The percentage of the expected number (7500 (75%), 5000 (50%), 500 (5%), 100 (1%), 50 (0.5%) and 25 (0.25%)) of deleted reads detected out of 10,000 identified by each pipeline. (F) Breakpoint mapping locations identified at each deleted read% level (75%–0.25%) in relation to the true breakpoint locations (highlighted in black).

**Figure 3:**
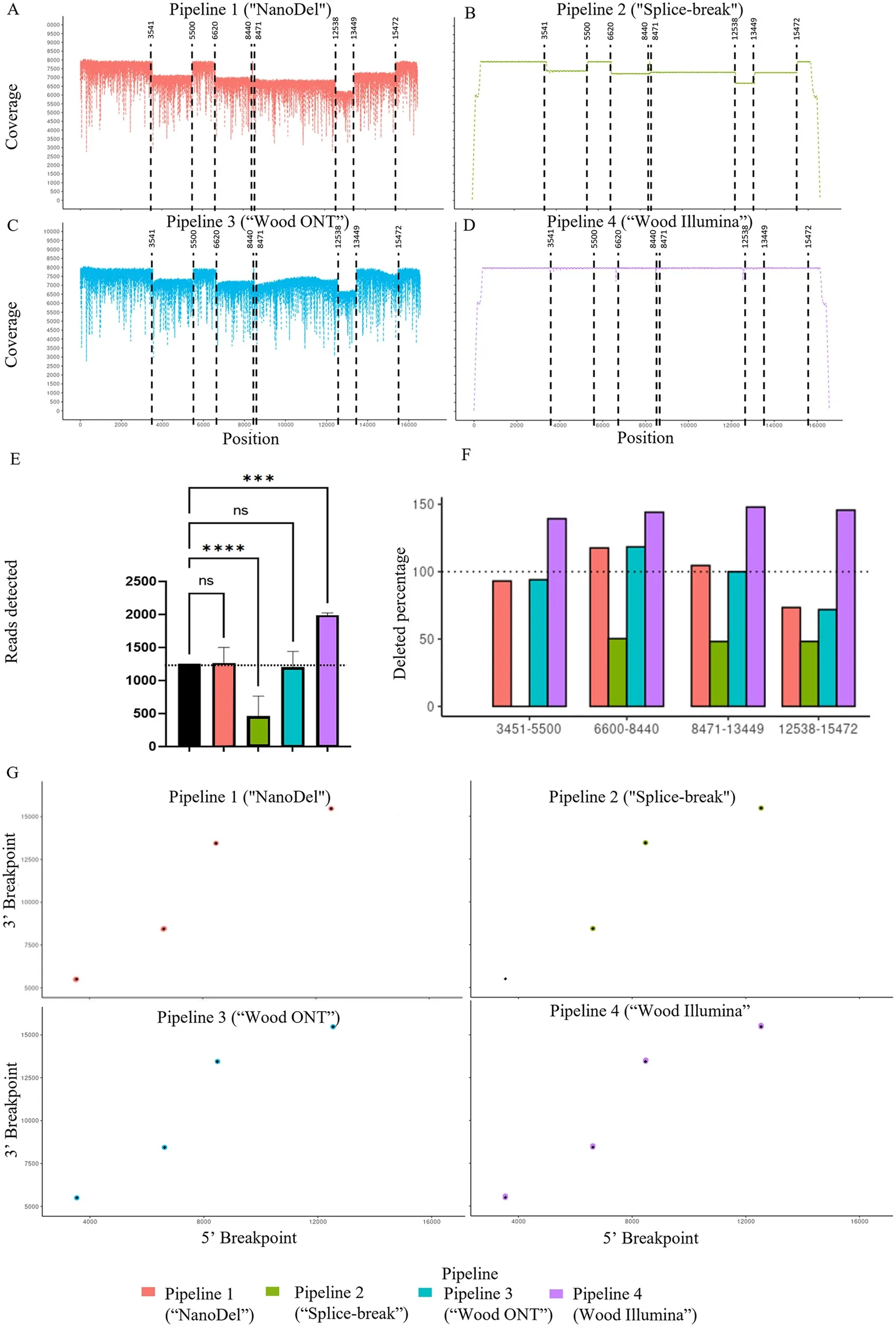
Analysis of artificial data set 2 that contains a mixture of deletions (i.e. between positions m. 8471_13449del, m. 6620_8440del, m. 3451_5500del and m. 2538_14572del), set at a read level of 10% and 90% wild type mtDNA. (A–D) The per position levels of coverage generated by Pipeline 1(“NanoDel”), Pipeline 2 (“Splice-break”), Pipeline 3 (“Wood ONT”), and Pipeline 4 (“Wood Illumina”), respectively. Breakpoint locations are denoted by black dashed lines. (E) The average number of deleted reads detected by each pipeline relative to the 1250 reads simulated (black bar). (F) The percentage deleted reads detected by deletion reads (so reads detected out of 1250*100). (G) Breakpoint mapping locations of each deletion identified in relation to the true breakpoint locations (highlighted in black). * = *P* < 0.05, ** = *P* < 0.01, *** = *P* < 0.001, and **** = *P* < 0.0001.

**Figure 4:**
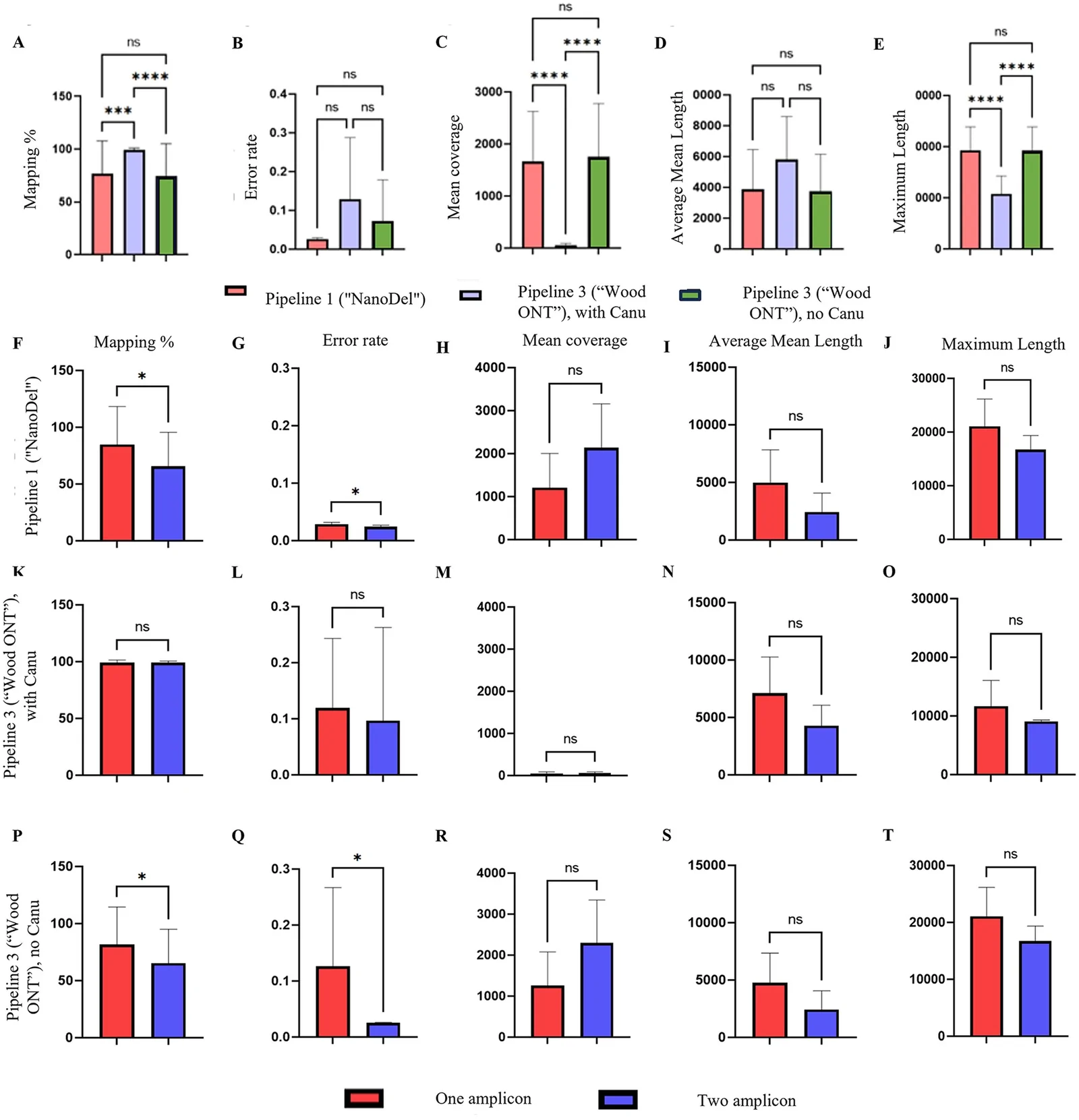
A comparison of post-alignment quality metrics: (A, F, K and P) The percentage of reads mapped to the reference genome, (B, G, L and Q) Error rate of mapped reads, (C, H, M and R) The average mean coverage, (D, I, N and S) Average read length, and (E, J, O and T) Maximum read length following the alignment of one and two-amplicon LR-PCR reads, analysed in combination (A–E, *n* = 16) or separately (F-T, *n* = 8), by Pipeline 1 (“NanoDel”), Pipeline 3 (“Wood ONT,” with Canu) and Pipeline 3 (without Canu) generated from patients with clinically diagnosed mitochondrial diseases. * = *P* < 0.05, ** = *P* < 0.01, *** = *P* < 0.001, and **** = *P* < 0.0001.

In addition to the differences between the post-alignment quality metrics, “NanoDel” (Pipeline 1) and “Wood ONT” (Pipeline 3) without Canu in combination with one-amplicon, rather than two-amplicon, LR-PCR best validated a known LSMD (m.6130_15055del) in KSS patient tissues (Fig. 5, Table S4A, and Supplementary results, available as supplementary data at *Bioinformatics* online). However, the average read % of m.6130_15055del was higher in the muscle, kidney and cerebellum using “NanoDel” and not correlated with the heteroplasmy % previously determined using Southern blotting (47%, 54% and 23%, respectively). Consequently, read percentages represent relative quantification and require validation (e.g. digital PCR or PCR-free approaches) before clinical interpretation as heteroplasmy levels.

**Figure 5:**
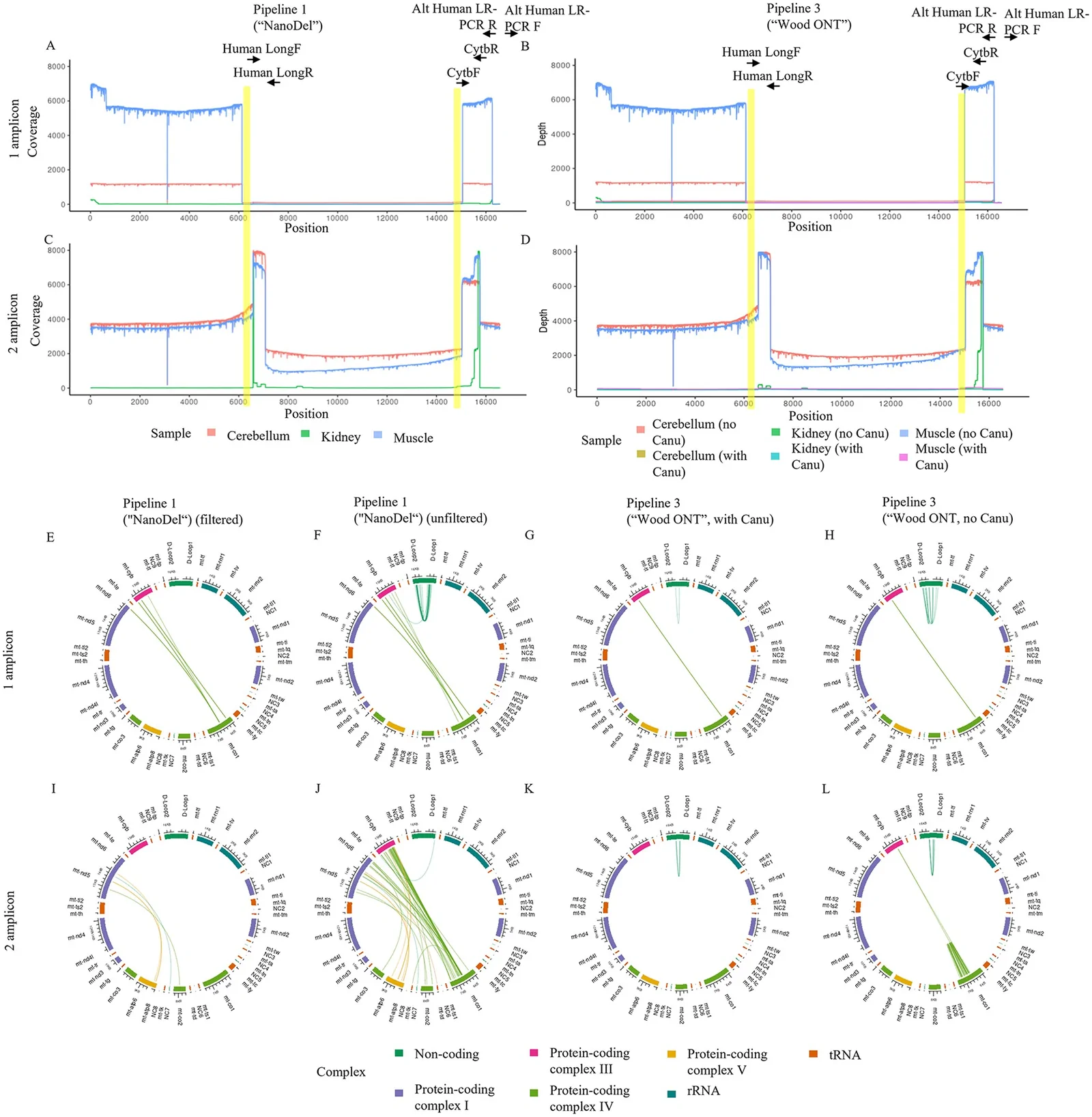
Analysis of coverage and deletion boundaries in different tissues of a KSS patient. (A–D) The levels of coverage displayed as read depth at each position for each sample amplified as either one (A, B) or two (C, D) amplicons, aligned with Pipeline 1 (“NanoDel,” A, C) or Pipeline 3 (“Wood ONT,” B, D). (E-L). Circos plots showing the 5’- and 3’-breakpoints of deletions following one (E-H) and two (I-L) amplicon LR-PCR aligned with Pipeline 1 (“NanoDel”) post-filtering (E, I) and unfiltered (F, J) and Pipeline 3 (“Wood ONT”) with (G, K) and without (H, L) Canu. The genomic links highlight the connected 5’- and 3’-breakpoints coloured by sample of detection, and key complexes across the mitochondrial genome are highlighted. Links proceed in a clockwise direction starting from the D-loop 1 region. Putative G4-structure forming regions are highlighted in yellow.

As the two-amplicon LR-PCR did not detect the known deletion (m.6130_15056del) in the KSS samples after filtering with “NanoDel” (Pipeline 1) or at all with “Wood ONT” (Pipeline 3), subsequent analyses of the other mitochondrial disease samples focussed on “NanoDel” (Pipeline 1) and “Wood ONT” (Pipeline 3) using just the one-amplicon LR-PCR. “NanoDel” (Pipeline 1) and one-amplicon LR-PCR validated known information obtained using traditional methods but also yielded new insights into LSMDs in the other mitochondrial disease patients, consistent with, or even in the absence of, primary diagnosis (Fig. 6, Table S4B, and Supplementary results, available as supplementary data at *Bioinformatics* online).

**Figure 6:**
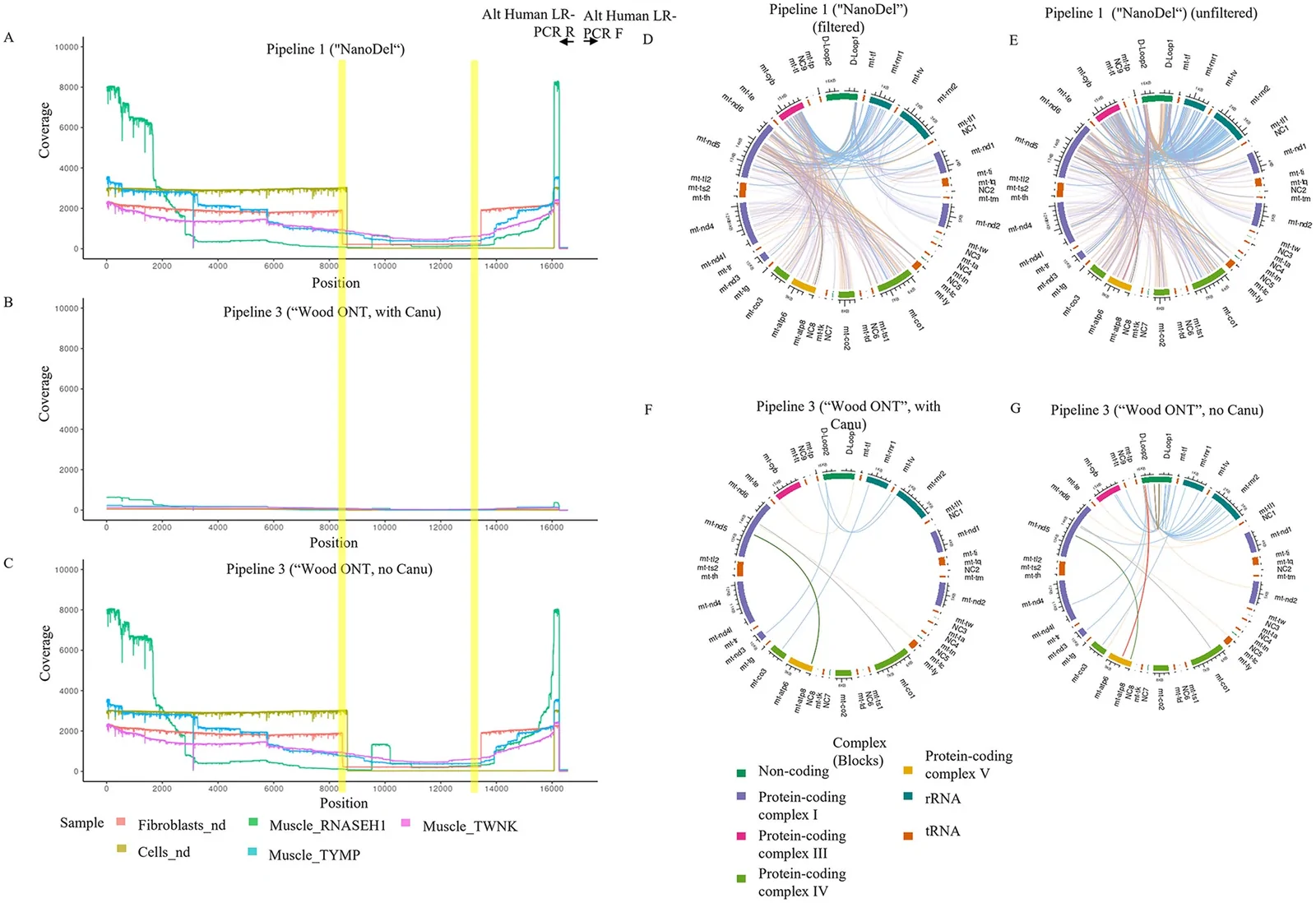
Analysis of coverage and deletion breakpoints in five samples clinically diagnosed with mitochondrial disease. (A–C) Shows the coverage at each position for each sample generated with Pipeline (“NanoDel,” A), and Pipeline 3 (“Wood ONT“) with Canu, (B) and without Canu (C), respectively. (D–G) Shows the 5’- and 3’-breakpoints of deletions identified by each pipeline in each sample as a circos plot following one-amplicon LR-PCR aligned with Pipeline 1 (“NanoDel”) post-filtering (D) and unfiltered (E) and Pipeline 3 (“Wood ONT”) with (F) and without (G) Canu. The genomic links highlight the connected 5’ and 3’ breakpoints coloured by sample of detection, and key complexes across the mitochondrial genome are highlighted. Links proceed in a clockwise direction starting from the D-loop 1 region. Putative G4-structure forming regions are highlighted in yellow.

Specifically, “NanoDel” (Pipeline 1) in combination with one-amplicon LR-PCR determined that for the three KSS samples 4/38 and 3/38 loci harboured 5’ and 3’-breakpoints, respectively with the highest 5’-breakpoint counts observed in mt-co1 (10), mt-cyb (1), mt-nd6 (1), and nr2 (1), and 3’-breakpoint counts observed in mt-cyb (9), mt-nd6 (3) and mt-rnr2 (1). Dividing the breakpoint count by locus length to calculate breakpoint density did not affect which loci were the most frequently deleted. In the other five mitochondrial disease tissue samples, 22/28 and 23/38 loci harboured 5’ and 3’-breakpoints, respectively. The top three 5’-breakpoint were observed in mt-co1 (103), mt-rnr1 (55), mt-rnr2 (50), and 3’-breakpoints were observed in mt-nd5 (155), mt-cyb (148), and mt-nd6 (61), respectively. However, 5’-breakpoint density was highest in mt-ts1, mt-tc, and mt-tl1, and 3’-breakpoint density was highest in mt-cyb, mt-nd6 and m-tl2, suggesting locus length can have an influence breakpoint density. Overall, no correlation was observed between breakpoint density and %GC content. The breakpoints identified for m.6130_15055del in the KSS samples and m.8469_13446del in fibroblast_nd, which occurred at ≥50% of reads in filtered “NanoDel” (Pipeline 1) data lie adjacent to or within repeat sequences and putative G4 structure-forming sequences, and are associated with a range of healthy and pathological tissues (Fig. 6, Table S5, and Supplementary results, available as supplementary data at *Bioinformatics* online).

## 4 Discussion

### 4.1 Development, validation, and potential clinical application of “NanoDel”

In this study, we first developed and then evaluated the ability of four pipelines to accurately detect the location and quantify the read% of LSMDs using artificial data sets. These pipelines included both DNA and splice-aware RNA alignment methodologies and were designed based on either short read (Illumina) or long-read (ONT) data sets. The pipelines selected for comparison against “NanoDel” (Pipeline 1, ONT splice-aware alignment, this study) were: “Splice-Break” (Pipeline 2, Illumina splice-aware alignment, Hjelm *et al.* 2019), “Wood ONT” (Pipeline 3, ONT DNA alignment, Wood *et al.* 2019) and “Wood Illumina” (Pipeline 4, Illumina DNA alignment, Wood *et al.* 2019). Splice-Break (Hjelm *et al.* 2019) was chosen as a comparator as it uses Illumina SRS in combination with a non-traditional splice-aware RNA alignment strategy that is not negatively affected by the presence of large-scale deletions. It has been shown previously to improve LSMDs calling accuracy compared to e.g. MitoDel (Bosworth *et al.* 2017). “Wood ONT” (Pipeline 3) and “Wood Illumina” (Pipeline 4) were chosen as they were being tested in a UK diagnostic setting and use either Illumina SRS or ONT LRS, respectively, in combination with a more traditional DNA alignment strategy at the inception of the study (Wood *et al.* 2019).

Additional pipelines for detecting large-scale deletions have been published since the inception of this study, including MitoSalt (Basu *et al.* 2020) and Sniffles2 (Smolka *et al.* 2024). MitoSalt, which also uses a splice-aware aligner HISAT2, has shown performance comparable to Splice-Break for LSMD detection using short-read data (Basu *et al.* 2020), and was recently demonstrated to detect LSMDs in long-read datasets (Barresi *et al.* 2025). In addition, MitoSalt can infer the presence of duplications in mtDNA, and when used in combination with Mitopore (Dobner *et al*. 2024), can also detect SNVs and haplogroups accurately and sensitively (Barresi *et al.* 2025). Sniffles2 is a general-purpose structural variant caller for LRS capable of detecting a broad spectrum of genomic rearrangements, including deletions, duplications, inversions, and translocations (Smolka *et al.* 2024), although it has not yet been evaluated for mtDNA analysis. Given these additional capabilities, using an additional set of one amplicon primers that cover the currently missing 304 bp in the mt-d-loop to capture full mtDNAs, together with a systematic comparison of MitoSalt, MitoPore and Sniffles2 with “NanoDel” represents important direction for future work. We demonstrate that our pipeline, “NanoDel” (Pipeline 1), which calls LSMDs from long-read data using a splice-aware alignment approach, offers improvements in accuracy and sensitivity over the other publicly available tools tested in this study. This is true in terms of both breakpoint location and read% quantification of mtDNA4977 (m.8471_13449del, Yusoff *et al.* 2020) and mixed populations of deletions, including at low levels, in artificial datasets. “NanoDel” (Pipeline 1) identified the correct breakpoint locations and number of reads for all the LSMDs. “Wood ONT” (Pipeline 3, DNA alignment) performed most similarly to “NanoDel” (Pipeline 1). This may be in part due to two factors: their use of a shared alignment tool ‘Minimap2‘(Li 2018; albeit using different settings, with “NanoDel” making use of the splice preset settings); and the fact that they both analysed the same style of long-read data compared to the other pipelines analysing ‘short-read style’ simulated data. In comparison, both “Splice-break” (Pipeline 2, Illumina RNA alignment) and “Wood Illumina” (Pipeline 4, DNA alignment) could not identify the LSMDs at the lowest 0.25 read% levels (“Splice-break”), or identified false positives (“Wood Illumina”), and were the least able to accurately quantify the correct number of reads containing LSMDs. Both these pipelines made use of short Illumina-style reads.

While these findings align with separate prior reports emphasizing the importance of using splice-aware aligners in LSMDs calling (Hjelm *et al.* 2019) and the potential utility of ONT sequencing in mitochondrial genomics (Frascarelli *et al.* 2023, Vandiver *et al.* 2023, Wood *et al.* 2019), they also highlight the potential strength of combining splice-aware alignment methodologies with LRS (i.e. “NanoDel”) in LSMDs calling. Consequently, we wanted to deploy “NanoDel” (Pipeline 1), and “Wood ONT” (Pipeline 3) as a comparator, to investigate LSMDs in clinical samples from patients diagnosed with mitochondrial disease and varying amounts of LSMDs and compare with the information obtained using traditional techniques such as Southern blotting.

In doing so, we found that “NanoDel” in combination with one-amplicon PCR, outperformed the alternative “Wood ONT” in terms of mapping and error rate (when used without Canu) when investigating LSMDs in different tissues from mitochondrial disease patients. “NanoDel” in combination with one-amplicon LR-PCR was able to identify the previously observed LSMD (m.6130_15056del) (Poulton *et al.* 1989, 1993, 1995) in KSS patient samples in a tissue specific manner. Furthermore, “NanoDel” was not only able to identify previously observed LSMDs in different tissues from five other patients diagnosed with mitochondrial disease, but also revealed additional, previously unreported LSMDs information. This demonstrated that “NanoDel” has power to identify LSMDs in different sample types (tissues/diseases), including more complex biological samples (such as those without a known genetic cause), with significant potential in a clinical setting. Validation using a larger more diverse cohort of patient samples would confirm this and is warranted.

Our findings demonstrated that “NanoDel,” in combination with one-amplicon LR-PCR, outperformed our two-amplicon approach. This is consistent with an original report that detailed the development and potential benefits of the one-amplicon LR-PCR approach over multi-amplicon approaches, including increased discriminating power against nuclear mitochondrial DNA segments (NUMTs), more even coverage, and increased LSMD detection resolution by limiting amplicon dropout (Zhang *et al.* 2012). NUMTs are pieces of mtDNA that have been transferred to and integrated into the nuclear genome and can cause false positives during PCR amplification. Consistent with the original report, we also showed that NUMTs (or DNA) were not amplified in our rho-zero mtDNA depleted controls with either one- or two-amplicon LR-PCR (Fig. S2, available as supplementary data at *Bioinformatics* online). Further, we showed the coverage was more even in our samples, with one rather than two-amplicon LR-PCR. We found that LSMDs were only identified within individual amplicon boundaries for both LR-PCR approaches. Furthermore, the inflated number of reads within overlapping regions relative to benchmark coverage affected the read% of LSMDs following two-amplicon PCR (see also Fig. S1, available as supplementary data at *Bioinformatics* online for further explanation). These findings suggest prior reports detailing mtDNA variants following the use of multi-amplicon PCR approaches, of which there are numerous examples including (but not restricted to) colorectal cancer (Aikhionbare *et al.* 2004), pancreatic cancer (Lam *et al.* 2012) and hypertension (Elango *et al.* 2011), could be incomplete and contain inaccuracies and should be interpreted with caution.

While the clinical application of ONT sequencing for mtDNA variants (including LSMD detection) has been proposed previously (Wood *et al.* 2019), recent improvements to the ONT chemistry now routinely generate base-calling quality scores of Q20+. This, taken together with “NanoDel”’s ability to yield a sensitive and accurate, and thus a comprehensive view of the LSMDs breakpoints/landscape without a priori information in individual samples (in combination with one-amplicon LR-PCR), offers improvements over traditional methods in this context. However, the limited of correlation in read% between “NanoDel” (and “Wood ONT”), in combination with one-amplicon LR-PCR, and the heteroplasmy measures obtained using traditional methods, could be due to the preferential amplification of shorter fragments during PCR in our samples (Kopsidas *et al.* 2000). While such preferential amplification has been shown to be minimised via the use of LR-PCR (Kopsidas *et al.* 2000), its impact could be verified in our samples by determining the ratio of deleted molecules to wild type molecules using digital PCR. Thus, for now, the read percentages should not be interpreted as heteroplasmy rates directly. Alternative PCR-free methods, e.g. purifying mitochondria/mtDNA prior to sequencing or WGS in combination with adaptive sequencing (where ONT pores screen for ‘expected’ sequences in silico, and reject strands that do not meet the expected requirements) may be used to increase sequencing yield of an intended target like mtDNA (Martin *et al.* 2022), and may more accurately quantify heteroplasmy. However, these methods also present challenges (e.g. Herbert *et al.* 2025). It can be difficult to obtain suitable yields of purified mtDNA (often due to its fragility), and WGS can generate poor coverage as the probes are not explicitly designed for mtDNA.

### 4.2 Mechanistic insights and broader implications

It has long been proposed that sequence-specific features, such as repeat sequences, can predispose certain regions of mtDNA to deletion events/hotspots. Indeed, most deletions occur between perfect repeat sequences (68%; class I deletions), imperfect repeat sequences (12%; class II deletions), or do not occur between repeat sequences (20%; class III deletions). Class I/II deletions are thought to be caused by replication error and slipped mispairing through two repeats, while class II/III deletions are thought to be caused by repair, mediated by ds-breaks (Chen *et al.* 2011). However, recently a non-uniform distribution of breakpoints has been described that cannot be explained by direct repeats alone. It is rather due to the major arc of the mtDNA forming a single-stranded secondary structure, which brings certain regions of the mtDNA closer together into a large-scale hairpin-like loop, with a “contact zone” between 6–9 kb and 13–16 kb (with a centre around 11 kb), which causes higher deletion activity (Shamanskiy *et al.* 2023). Using “NanoDel,” we identified the highest counts of 5’-breakpoints in the gene mt-co1 and 3’-breakpoints in the genes mt-cyb, mt-nd5 and mt-nd6 in relation to other loci. Our sample size is too small to study the distribution of breakpoints comprehensively; however, we note that all these genes occur within the recently described “contact zone” (Shamanskiy *et al.* 2023), and so further study is warranted to confirm whether this could be the driver.

It is possible that other structural features (e.g. G4s) could also predispose regions of the mtDNA to deletion events/hotspots. G4s are four-stranded nucleic acid structures formed by guanine-rich sequences in DNA and RNA. They are non-canonical structures, meaning they deviate from the typical double-helix structure of B-form DNA. G4s are formed by Hoogsteen hydrogen bonding of four guanine bases in a planar arrangement called a G-quartet, stabilised by a cation (e.g. potassium), which then stack on top of each other to form the four-stranded structure. They can exist in different topologies depending on the orientation of the guanines within the quartet, and can be found in various genomic locations, including telomeres, promoters, untranslated regions of mRNA, and mtDNA (Huppert and Balasubramanian 2007, Rhodes and Lipps 2015).

Consequently, we looked at the location of two LSMDs breakpoints relative to repeat regions, putative G4s, and the recently described “contact zone.” These features have long been implicated in the formation of mtDNA deletions through slipped-strand mispairing, and replication stalling mechanisms (Phillips *et al.* 2017). Here, identical, or highly similar DNA sequences are mispaired during replication by the mtDNA polymerase, which slides along the template leading to the removal of the region between the repeats. Furthermore, replication may stall at non-canonical DNA structures such as G4s (or the newly identified large-scale hairpin-like loop), causing it to become damaged through ds-breaks. This could be mediated by microhomology-mediated end joining (MMEJ), one of the main DSBR repair mechanisms in the mtDNA, which has been implicated in the formation of LSMDs. Since MMEJ requires micro-homologous regions of 5–22 bp in length, these imperfect repeat sequences may have determined the specific location where these LSMDs formed (McVey and Lee 2008, Tadi *et al.* 2016). Thus, a plausible mechanism for LSMD formation in cells/tissues involves genomic instability caused by secondary structures, replication fork slippage, transcription arrest, leading to the introduction of double-strand breaks (DSBs) via Endonuclease G that are then repaired via MMEJ, ultimately resulting in the formation of LSMDs (Dahal *et al.* 2022). This might be particularly true in the context of mtDNA, which is very G-rich and has fewer ROS-protective mechanisms and is therefore prone to destabilization and the formation of G4s (Aleksič *et al.* 2025).

The two LSMDs investigated more closely in this study m.6130_15055del in the KSS samples and m.8469_13446del in fibroblasts_nd were adjacent to or within either perfect or imperfect repeats, or potential G4-forming motifs, identified using QGRS mapper and AF3. In both cases scores were low, although AF3 suggests certain putative G4 sequences identified with QGRS Mapper are more likely to form in the presence of K + ions. This is perhaps as expected, since metal cations coordinate the G-tetrads leading to the stabilisation of G4s (Phan 2010). While AF3 represents a significant advancement in macromolecule (particularly protein) structure prediction, its ability to predict structures is influenced by the availability of accurate experimental and training structures and/or evolutionary information, such as homologous sequences e.g. from the Protein Databank (PDB, Berman *et al.* 2000) and other databases. While there are some 70 G4-structures in the PDB, none are from mtDNA, which may have limited the ability of AF3 to predict structures with confidence for the LSMDs m.6130_15055del in the KSS samples and m.8469_13446del in fibroblasts_nd.

While it is possible that some G4-structures are more likely to form than others, based on specific sequences, the balance between ds-DNA and G4-stability and the chemical environment at a particular point in time, other “spare” G-tracts may to form into G4s, and may serve to mitigate the effects of the damaged G4s, preserving G4-function under stress conditions (Aleksič *et al.* 2025). The predicted G4-sequences in this study, yet unresolvable by AF3, might represent such “spare” regulatory elements. As more G4, especially mtDNA-G4, structures become available the ability of AF3 to predict structures accurately is set to increase and should shed light on the authenticity/importance of the other putative G4-sequences identified by QGRS Mapper in the future.

However, it is also worth noting that the m.6130_15055del in the KSS samples and m.8469_13446del in fibroblasts_nd. (and, therefore, their associated repeat sequences and putative G4s) also occur within the “contact zone” identified by Shamanskiy *et al.* (2023). While Shamanskiy *et al.* (2023) showed that the structure of this “contact zone” is what’s responsible for higher than expected deletion activity (rather than the direct repeats themselves), they did not explore the role of G4s.

Nonetheless, these findings could suggest that the mtDNA deletions, including those detected by “NanoDel,” are not random, and that their formation is guided by both inherent sequence architecture and interactions with other factors. This includes ions that might be modulated within the mitochondria by the cellular microenvironment, thus contributing to our understanding of mtDNA instability in both health and disease. Interestingly, the two LSMDs investigated in more detail in our mitochondrial disease samples (this study) overlap with deletions observed in aging, metabolically active, and cancer tissues, as well as in neurodegenerative diseases, suggesting a shared vulnerability landscape shaped by mtDNA sequence motifs and structural constraints, and may support the concept of “deletion hotspots” (Schon *et al.* 1989) as a unifying feature of mitochondrial dysfunction across multiple pathological states.

## Supplementary Material

btag684_Supplementary_Data

## Data Availability

The raw sequencing data underlying this article are available in the NCBI Sequence Read Archive (SRA) under BioProject accession code PRJNA1369153 (https://www.ncbi.nlm.nih.gov/bioproject/1369153). Simulated benchmark datasets used for validation are included directly within the software repository.
